# Nonreplacement therapy for hemophilia in low-income countries: experience from a prospective study in Ivory Coast

**DOI:** 10.1016/j.rpth.2022.100033

**Published:** 2022-12-28

**Authors:** Catherine Lambert, N’Dogomo Meité, Gustave Koffi Kouassi, Alexis Silué Dohoma, Sara Adélaide Bognini Akou, Ibrahima Sanogo, Cedric Hermans

**Affiliations:** 1 Hemostasis and Thrombosis Unit, Division of Haematology, Cliniques Universitaires Saint-Luc, Brussels, Belgium; 2 Division of Clinical Haematology, Centre Hospitalier Universitaire de Yopougon, Abidjan, Ivory Coast

**Keywords:** emicizumab, hemophilia A, innovative therapies, Ivory Coast, low-income countries

## Abstract

**Background:**

Hemophilia management has fundamentally evolved over the last decades with the development of ground-breaking therapies. Because of their mode of action and biochemical properties, these innovative therapies that are available in developed countries could be readily implemented among people from low-income countries who are either not or inadequately treated with clotting factor concentrates (CFCs).

****Objectives**:**

We aimed at evaluating the impact of prophylaxis with emicizumab, a bispecific monoclonal antibody mimicking the FVIII activity administered subcutaneously, among boys with severe hemophilia A (HA) from the Ivory Coast, where access to CFCs is limited to humanitarian aid.

**Methods:**

We prospectively collected data on the implementation and outcomes of prophylaxis with emicizumab, in 33 Ivorian boys aged 2 to 13 years with severe HA (with and without inhibitors). Bleeds, CFC consumption, quality of life and satisfaction of the patients and their parents were assessed.

**Results:**

Overall, 12 months after initiating emicizumab, a 99% reduction in bleeding rates was observed, with a raise from 18% to 100% of boys having zero spontaneous joint bleeds. Three boys required a single FVIII infusion following a traumatic bleed. Health-related quality of life measures significantly improved, and perception of treatment efficacy was positively rated in children and parents. Acceptance, tolerance, and adherence were excellent. Emicizumab was instrumental in successfully implementing uninterrupted, highly efficacious, and well-tolerated prophylaxis in 72% of the Ivorian children aged ≤ 13 years identified with severe hemophilia A.

**Conclusion:**

These data illustrate how innovative and disruptive nonreplacement therapies that are already accessible in developed countries could potentially provide equity in care by profoundly and rapidly modifying hemophilia burden with a magnified impact in low-income countries.

## Essentials

• New hemophilia treatment options are currently mainly available in high-income countries.
• A majority of people with hemophilia worldwide still have no access to appropriate care.
• We studied how access to emicizumab modified the burden of hemophilia in boys from Ivory Coast.
• The impact of innovative hemophilia therapies is magnified in low-income countries.

## 1 Introduction

Successful investments and achievements in basic and clinical research have led to innovative therapies that are dramatically improving the prognosis and quality of life of patients with rare diseases, mainly in high-income countries. Hemophilia is among the rare diseases whose management has fundamentally evolved over the past few decades thanks to the constant development and implementation of new treatment options. [1]

Hemophilia is a rare inherited bleeding disorder characterized by partial to complete deficiency in either clotting factor VIII (FVIII) (Hemophilia A) or factor IX (FIX) (Hemophilia B). [2] Depending on disease severity, people with hemophilia (PWH) experience bleeding manifestations, primarily affecting joints, muscles, and soft tissues, but also internal organs with possible life-threatening consequences. [2,3] In the absence of efficient treatment, these bleeding episodes can lead to hemophilic arthropathy, chronic pain, disability, impaired health-related quality of life (HR-QoL), and even premature death. [4]

In PWH exhibiting a severe bleeding phenotype, the World Federation of Hemophilia (WFH) recommends regular preventive hemostatic treatment as a standard of care. [5] During the past four decades, this prevention has consisted in regular intravenous (i.v.) infusions of clotting factor concentrates (CFCs), ideally initiated very early in life, which were initially derived from plasma and later produced by biotechnology. The need for i.v. administration of these CFCs, their rapid clearance from the blood after administration, and their immunogenicity with concurrent development of neutralizing antibodies, mainly in severe hemophilia A (HA), represent major obstacles of CFC-based replacement therapy of hemophilia. [6]

Therapies not based on i.v. substitution of the missing FVIII or FIX but using alternative technologies have recently been developed and validated. These hemostatic therapies either mimic the defective clotting FVIII or rebalance the coagulation process by targeting coagulation inhibitors. Because of their mode of action and biochemical properties, these new agents can be administered subcutaneously to PWH with and without neutralizing antibodies against CFCs, at a low frequency of administration and without significant risk of immunogenicity. These ground-breaking therapies have recently demonstrated their potential to radically modify the management of PWH treated with CFCs, including those with inhibitors. [7,8] An even more promising aspect is that these new therapies, because of their favorable profile, can be implemented readily among the hundreds of thousands of HA patients who are currently not being treated or are inadequately with CFCs worldwide. [1]

We herein report the experience of implementing treatment with emicizumab, a subcutaneously administered humanized bispecific mAb mimicking the function of FVIII, to treat young boys with severe HA in the Ivory Coast. These data demonstrate the huge potential of such revolutionary therapeutic options to treat rare diseases like hemophilia in resource-constrained countries.

## 2 Context

This initiative took place in a Sub-Saharan African country where access to diagnosis and appropriate hemophilia management remain challenging and where CFCs available in limited amounts are exclusively issued from the WFH Humanitarian Aid Program. Over the last years, awareness and management of hemophilia have gradually improved in the Ivory Coast thanks to WFH initiatives including an active twinning program and the initiation of a low-dose and low-frequency prophylaxis program in children with donated CFCs. [9] This eventually led to the recognition of hemophilia as a major health issue by the local health authorities in 2021. Following a coinvestment agreement between F. Hoffmann-La Roche Ltd and the Ivorian government, access to emicizumab was made possible in April 2021. The drug was granted a temporary marketing authorization, being provided free of charge in limited but sufficient amounts to treat children in priority. In June 2021, 46 children aged ≤13 years with severe HA had been identified across the country. All were regularly followed-up at the hemophilia treatment center (HTC) of Yopougon in Abidjan, the only HTC in the country.

## 3 Methods

For the purpose of rigorous and objective documentation, a prospective study was set up with the aim of collecting data on implementation and outcomes of prophylaxis with emicizumab among Ivorian boys with severe HA. The inclusion criteria were as follows: boys aged 2 to 13 years with severe HA, with or without inhibitors, regularly followed at least for 12 months at the HTC of Abidjan, and whose parents agreed to participate and were willing to perform prophylaxis. Emicizumab was delivered at the HTC in Abidjan and initially administered at the HTC or the proximity hospital. Following education of the parents, home therapy was allowed. A rescue dose of CFC (Fc-rFVIII-efmoroctocog alfa) was provided to each participant to treat potential breakthrough bleeding episodes without any delay. Data on bleeds, FVIII consumption, as well as number of days of hospitalization and absence from school were collected both from patients’ logbooks and during the follow-up visits. Data were gathered retrospectively 12 months before inclusion and prospectively during a 12-month follow-up period. Activated partial thromboplastin time (aPTT) was measured at inclusion (T0) and 2 months after initiating emicizumab to confirm the *in vitro* activity of the medication and treatment compliance. HR-QoL data were assessed for children aged >4 years at emicizumab initiation (T0) and 6 months later (T1) using the Ivorian version of the Canadian Haemophilia Outcomes-Kids’ Life Assessment Tool (CHO-KLAT)_2.0_ [10] (proxy reports only) and the EQ-5D-5L (proxy reports and self-reports in children aged >8 years). The patient’s treatment satisfaction was evaluated at T1 using the self-report measure patient global impression of change (PGIC) in all parents and in children aged >8 years. The study was approved by the local ethics committee (001-22/MSHPCMU/CNESVS-km) and registered in ClinicalTrials.gov (NCT05279924). All parents signed an informed consent.

### 3.1 Statistical analysis

All analyses were performed using the JMP Pro software Version 14.3.0 (100 SAS Campus Drive Cary, USA). Continuous variables were expressed either as means and standard deviations or as medians and ranges and compared between groups using the Wilcoxon rank-sum test. Nonparametric paired tests were used to compare 2-time points. Categorical variables were expressed as counts and percentages and compared between groups using the Pearson chi-squared test. Statistical significance was set at *P* <0.05.

### 3.2 Role of the funding source

This study did not receive any financial support.

## 4 Results

### 4.1 Demographics

Overall, 37 boys aged 2 to 13 years with severe HA and followed-up at the HTC of Yopougon in Abidjan were identified as potential participants. Among them, 33 were enrolled and started prophylaxis with emicizumab (3 mg/kg/week for 4 weeks followed by 6 mg/kg/28 days [11]) between July and October 2021. A whole vial of emicizumab was administered with a dose rounded (±2.5 kg) as close as possible to the weight. Reasons for exclusion were parental refusal (n = 2), lack of adherence during the 12 months before screening (n = 1), and overweight (n = 1, aged 12 years and on low-dose prophylaxis with CFCs, as the amount of emicizumab was limited). The chosen treatment regimen allowed for fewer visits to the HTC as half of the participants lived in remote areas. All participants were followed prospectively at the HTC every 4 weeks for the first 6 months. At the end of this period, visits interval could be spaced at 2 months in participants whose parents were able and willing to inject emicizumab. The demographic characteristics of the study population are detailed in Table 1. Of note, at inclusion, 42% of patients were on the low-dose prophylaxis program with Fc-rFVIII issued from the WFH Humanitarian Aid Program for 3 years. However, because of interruptions in supply and donations during the COVID-19 pandemic, prophylaxis was paused on several occasions in the year before inclusion, and some adherence issues should also be mentioned.

**Table 1:** Demographic characteristics of the 33 boys with severe hemophilia A at inclusion (T0).

Median age [range]	8 y [2-13 y]
Median weight [range]	24 kg [11·7-40 kg]
Ethnicity	Black African 100% (n = 33)
Presence of inhibitors	6% (n = 2)
Treatment with factor VIII	
Low-dose and low-frequency prophylaxis^a^	42.2% (n = 14)
On demand	54.5% (n = 18)
Previously untreated patient	3% (n = 1)
Place of residence	
District of Abidjan	51% (n = 17)
Outside Abidjan	52% (n = 16)
Mean annual consumption of FVIII	7690 IU
Median annual consumption of FVIII [range]	6000 IU [0-22750 IU]
Median ABR [range]	6 [1-19]
Median ASJBR [range]	3 [0-12]
Proportion of boys with ABR at 0	0%
Proportion of boys with ASJBR at 0	18%
Proportion of boys having a target joint	21.2%
Number of target joint(s) (mean)	0·3 [0-3]
Median annual days off school [range]^b^	1 [0-10]
Mean annual days off school (SD)^b^	2.4 (2.9)
Median annual hospitalization days [range]	0 [0-1]

ABR, annual bleeding rate; ASJBR, annual spontaneous joint bleeding rate.

a Low-dose and low-frequency prophylaxis with 20 IU Kg^−1^ per week of Fc-rFVIII.

b Among the 26 school-age children.

Two boys had an inhibitor at inclusion. The first, aged 12 years, with a persistent inhibitor for 2 years, at a titre of 2.5 BU at T0 (peak titre at 2.5 BU), was on low-dose prophylaxis motivated by the frequency of bleeding and the high number of days missed at school. No anamnestic response occurred under prophylaxis. The second, aged 11 years, with a long-standing inhibitor for 5 years, at a titre of 3.2 BU at T0 (peak titre of 6.8 BU), was treated on demand with recombinant activated FVII. Neither of the two had benefited from immune tolerance therapy.

### 4.2 Prophylaxis outcomes

**Figure 1:**
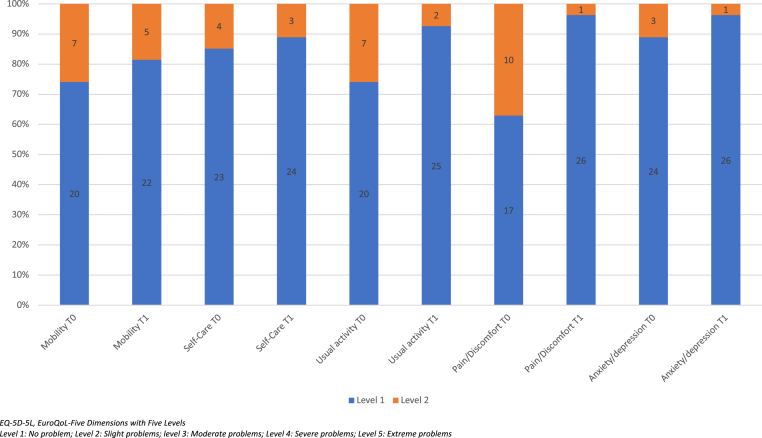
EQ-5D-5L frequencies and proportions at T0 and T1 reported by dimension and level by the parents of children aged >4 years (n = 27)

**Figure 2:**
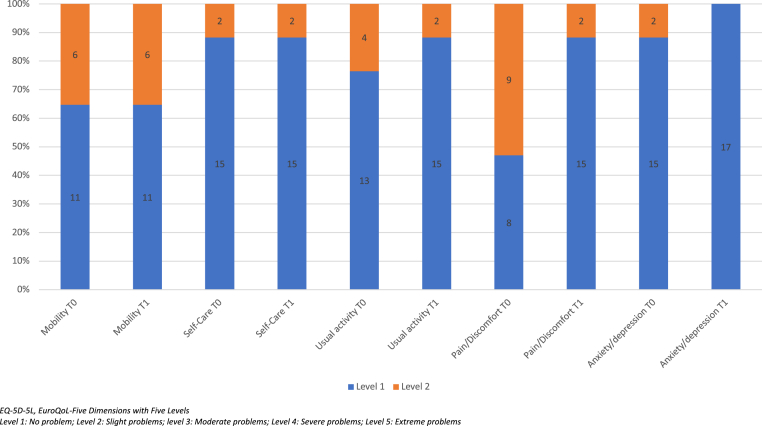
EQ-5D-5L frequencies and proportions at T0 and T1 reported by dimension and level by the children aged >8 years (n = 17)

The outcomes of emicizumab prophylaxis are summarized in Tables 2 and 3. After 12 months, a dramatic 99% reduction of the estimated annualized bleeding rate (ABR) was observed in the study population. The proportion of boys with no spontaneous joint bleeds rose from 18% to 100%. Three boys, without inhibitors, experienced a traumatic bleed (skin and tongue wound) that was successfully treated using a single injection of Fc-rFVIII (1000 IU for two of them and 2000 IU for the last one). No hospitalization was required, and only two boys who experienced a bleed missed 2 and 1 days of school, respectively. Two months after the initiation of emicizumab, aPTT values significantly decreased below the upper limit of the normal in all participants (mean aPTT decreased from 65.6 sec at T0 to 26.2 sec after 2 months [*P* < 0.0001]). There was no biological nor clinical suspicion of neutralizing antidrugs antibodies as no participants displayed spontaneous bleeds. A significant improvement in HR-QoL was noted both in the EQ-visual analog scale (VAS) from the EQ-5D-5L and in the total scores of the Ivorian version of the CHO-KLAT_2.0_ (Table 3). With respect to the EQ-5D-5L results, a statistically significant difference in the pain dimension was observed for both the proxy questionnaire (*P* = 0·0043) (Figure 1) and the self-questionnaire (*P* = 0·0043) (Figure 2) between T0 and T1. No significant differences were found between the subgroups living close to the HTC and those living outside the Abidjan district for the following parameters: annual FVIII consumption (*P* = 0.08) and ABR (*P* = 0.08) at T2, CHO-KLAT total scores (*P* = 0.9), CHO-KLAT general question 1 (*P* = 0.16), and VAS score of EQ-5-D-5L (*P* = 0.27) at T1.

**Table 2:** Clinical outcomes of prophylaxis with emicizumab.

Measure	N	T0	T2	T2-T0 Mean (±SEM)	*P*
ABR (mean)	33	7.36	0.09	−7·3 (±0·8)	<0.0001
ASJBR (mean)	33	3.3	0.0	−3·3 (±0.6)	<0.0001
Number of target joint(s) (mean)	33	0.3	0.0	−0.3 (0.1)	0.0159
Annual FVIII consumption (mean)	33	7690	151	−7539 (±1270)	0.0001

T0: inclusion, T2: 12 months after inclusion. ABR, annual bleeding rate; ASJBR, annual spontaneous joint bleeding rate.

**Table 3:** Quality of life outcomes of prophylaxis with emicizumab.

Measure	N	T0	T1	T1−T0	*P*
EQ-VAS for parents	27	73.5	88.7	14.7 (±1.5)	<0.0001
EQ-VAS for boys >8 years	17	76.8	89.6	12.8 (±1.3)	<0.0001
CHO-KLAT total score for parents	27	65.8	78.7	15.5 (±1.0)	<0.0001
CHO-KLAT score general Q1 for parents	27	2.1	1.2	−0.9 (±0.3)	0.0022

The results of EQ-VAS and CHO-KLAT are expressed in mean values. T0: inclusion, T1: 6 months after inclusion. CHO-KLAT_2.0_, Canadian Haemophilia Outcomes-Kids’ Life Assessment Tool version_2.0_; Q1: General question1 of CHO-KLAT_2.0_ providing the global rating of bother by hemophilia.

**Figure 3:**
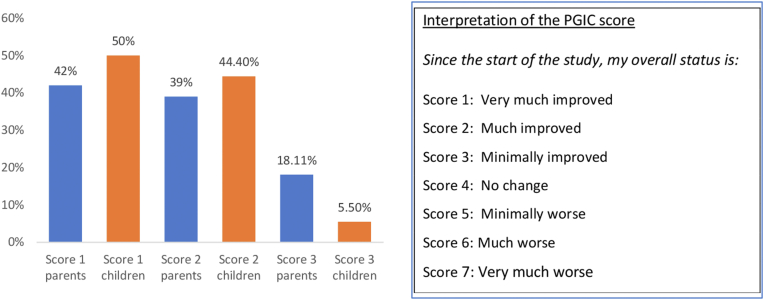
Proportion of the patient global impression of change (PGIC) scores of the parents (n = 33) and the children aged >8 years (n = 17) at T1

The parents’ (33) and childrens’ (17) perceptions about treatment efficacy were assessed at T1 using the PGIC questionnaire. A “considerable improvement” was reported by 42.4% of the parents and 50% of the boys, a “definite improvement that has made a real and worthwhile difference” was reported by 39.4% of the parents and 44.4% of the boys, and a “moderately better, and a slight but noticeable change” was reported by 18. 2% of the parents and 5.5% of the boys (Figure 3). The Pearson chi-squared test could not demonstrate a relationship between the PGIC response level and type of treatment before inclusion (on demand vs low-dose prophylaxis [*P =* 0.96]). Emicizumab tolerance was excellent, with no local reactions to infusions or other adverse events declared. No adherence issues (interruption or missed dose) and no losses to follow-up were reported. Parents gradually gained autonomy in injecting their children with emicizumab, making home prophylaxis possible in 63% of the population from 6 months after the inclusion.

## 5 Discussion

In low-income countries, where 70% of PWH live, very few individuals have access to adequate diagnosis and treatment, resulting in a high morbidity and mortality often early in life. The limited local resources available are indeed being allocated to multiple public health priorities, such as nutrition, immunization, sanitation, and treatment of infectious diseases, rather than to hemophilia. [12] In addition to very limited access to CFCs, poor awareness of hemophilia, scarce laboratory diagnostic capabilities, limited number of HTCs and experienced staff, costs related to travels to the HTCs, storage challenges, and expenses for disposables and CFCs infusions represent additional obstacles to hemophilia care and treatment in low-income countries. [12] The situation is even worse for patients with neutralizing antibodies against CFCs who do not have access to bypassing hemostatic agents or immune-tolerizing treatments.

Fortunately, the situation in the Ivory Coast has recently improved, with a significant number of PWH now having access to diagnosis and CFCs through the WFH twinning initiative and the expanded WFH Humanitarian Aid Program. The latter has actually made it possible to initiate a low-dose preventive treatment with CFCs in several children with severe hemophilia. However, the unavoidable need for regular i.v. CFC infusions, the need for high adherence, the risk of immunogenicity, and recurrent interruptions in supply are not compatible with the ambition to provide efficacious, well-tolerated, and persistent prevention of bleeds among all children with severe HA, especially in low-income countries.

The initiation of prophylaxis with a novel agent like emicizumab substantially improved hemophilia management in the Ivory Coast. As documented in our study, emicizumab was positively perceived by patients and their families and easy to implement, without major logistical investments. More importantly, it provided a high level of hemostatic efficacy with a significant improvement in quality of life and participation, without safety concerns. These real-life results in a low-income country are in line with those reported in Phase 3 studies conducted in developed countries. [13] emicizumab raised the proportion of children on prophylaxis from 30% to 72% among boys aged <13 years in the Ivory Coast, with a significant proportion carrying out home administration reducing herewith the indirect costs of the therapy.

The results of this study highlight the major direct and indirect benefits of implementing a highly innovative therapy of hemophilia in low-income countries. With its multiple desirable features, emicizumab can be used not only as a substitute to CFCs but more importantly as a first-line prophylactic hemostatic treatment for patients with no, limited, or difficult access to CFCs.

The situation in the Ivory Coast is unique as emicizumab is provided free of charge through a collaboration between the government and the pharmaceutical company producing the drug. The data collected in this study will hopefully promote the continuation of this program, expand to other countries, and allow access to sustainable therapy for the hemophilia community at a global level. At the present time, 15 boys with severe HA aged 13 to 18 years and three adults with a long-standing inhibitor have recently started prophylaxis with emicizumab in the Ivory Coast.

Beyond emicizumab, similar findings could likely be obtained in the future with other new treatment options of hemophilia such as rebalancing agents, improved formulations of CFCs, and possibly gene therapy that is currently being validated in developed countries. In low-income countries around the globe, these results should encourage more donations as those organized by the WFH [14] or ideally stimulate local funding of novel therapies of hemophilia.

Access to innovative therapies can however only be successfully implemented among those PWH who have an early and valid diagnosis of hemophilia and have been well-educated about their disease. This emphasizes the crucial role of promoting awareness of the disease, medical education, as well as laboratory diagnosis and active screening.

## 6 Conclusion

Emicizumab, a new treatment of hemophilia, successfully contributed to implementing uninterrupted, highly efficacious, and well-tolerated prophylaxis in most children with severe HA in the Ivory Coast. These data illustrate in a very original way how highly innovative and ground-breaking therapies currently accessible in developed countries can potentially provide equity in care globally by profoundly and rapidly modifying the burden of a rare disease with a magnified impact in low-income countries.
